# Differences in Mpox and Vaccinia Immunity Induced by Non-Replicating and Replicating Vaccinia-Based Vaccines

**DOI:** 10.3390/vaccines13050520

**Published:** 2025-05-14

**Authors:** Getahun Abate, Krystal Meza, Yinyi Yu, Chase Colbert, Anna Jaunarajs, Azra Blazevic, Daniel F. Hoft, Sharon E. Frey

**Affiliations:** 1 Division of Infectious Diseases, Allergy and Immunology, Saint Louis University, St. Louis, MO 63104, USA; 2 EMMES, Rockville, MD 20850, USA

**Keywords:** mpox, vaccinia, MVA-BN, Dryvax, replication-competent, immunity

## Abstract

**Background**: The recent global outbreak with clade IIb and the concurrent emergence of clade I mpox virus in Africa show that mpox is a challenging problem. MVA-BN induces low-level mpox-neutralizing antibody responses that wane rapidly. This study was conducted to compare the mpox immunity induced by a replication-competent smallpox vaccine and non-replicating MVA-BN. **Methods:** Stored sera (*n* = 302) and PBMCs (*n* = 244) collected pre-vaccination and at five post-vaccination time points in MVA-BN and six post-vaccination time points in Dryvax clinical trials were used. Antibody titers that neutralized at least 50% of mpox in cell culture were determined by the focus reduction neutralization test (FRNT) 50, and the mpox-specific T cell responses were measured using an IFN-γ ELISPOT assay. **Results:** The peak geometric fold rise (95% CI) (i.e., the maximum GMFR across all study visits) in the mpox FRNT50 for subcutaneous (SC) MVA-BN, intradermal (ID) MVA-BN, and Dryvax was 22.1 (8.3, 59.1), 18.5 (8.0, 43.1), and 245.8 (100.4, 601.6), respectively. The GMFR at day 180 post-vaccination for MVA-BN (SC), MVA-BN (ID), and Dryvax was 2.4, 2.7, and 64, respectively. The mean (95% CI) peak number of mpox-specific IFN-γ-producing SFCs was 127 (43.1, 238.3), 87.3 (46, 137), and 61.2 (44.3, 77.7) for MVA-BN (SC), MVA-BN (ID), and Dryvax, respectively. On day 180, the mean SFCs in the three groups decreased to 10.8 (−34.4, 3.8), 3.3 (−6.2, 18.6), and 2.2 (−9, 12.5), respectively. **Conclusions**: The peak mpox-neutralizing antibody titer was >10-fold lower in MVA-BN recipients compared to those who received a replication-competent smallpox vaccine, and the level at day 180 was >20 times lower in MVA-BN recipients. MVA-BN induced similar or higher T cell responses.

## 1. Introduction

Mpox is caused by the monkeypox virus (MPXV), a member of the Orthopoxvirus genus, which includes poxviruses that cause disease in humans and animals [1]. MPXV has two clades, clade I and clade II [2,3]. It is transmitted through close contact with an infected animal or human, infected fomites, or contact with secretions contaminated with the virus [4,5]. Prior to 2022, MPXV caused multiple sporadic outbreaks in Africa and one small outbreak in the USA in 2003 [5]. MPXV showed its potential to cause global outbreaks in the recent spread of clade IIb and clade I MPXV to countries outside of Africa. The global mpox outbreak with clade IIb that started in May 2022 caused more than 100,000 cases in 122 countries, including 115 countries where mpox was not previously reported [6]. In July 2022, the number of cases started to decline, but the outbreak continued to cause morbidity and mortality [7]. The exact reasons why the outbreak waned are unclear, but could be related to improved awareness and behavioral changes in the population at risk and the acquisition of vaccination- or infection-induced immunity [8]. In 2024, there were 5539 cases from the Region of the Americas and 2267 cases from the Western Pacific Region [7]. Concordantly, there was an emergence and spread of clade I MPXV in Africa. On 14 August 2024, the WHO declared that the mpox outbreak in Africa constituted a public health emergency of international concern (PHEIC) [9]. In 2024, 20 African countries reported 13,760 confirmed cases, including 60 deaths [7].

Smallpox vaccines provide protection against mpox. All widely known or approved smallpox vaccines are derived from vaccinia, and these vaccines are generally classified into replication-deficient and replication-competent vaccines based on their capacity to replicate in mammalian cells [1]. Modified vaccinia Ankara (MVA) from Bovarian Nordic (BN, Denmark), developed by passaging the Ankara strain of vaccinia more than 570 times in chick embryo fibroblasts, is the only non-replicating vaccine [1]. Examples of replication-competent smallpox vaccines include Dryvax, ACAM2000, the Aventis Pasteur smallpox vaccine (APSV), and Lister vaccine/LC16. During the smallpox eradication programs before 1972, Dryvax and the Lister strain were the vaccines used in the US, Europe, Africa, and Asia [10]. Progress to improve smallpox vaccination was stalled until 2002, when the threat of using smallpox as a bioterrorism agent increased. Several safety and immunogenicity studies on standard and diluted doses of smallpox vaccines were conducted in order to help strategize the use of available vaccines and improve national stockpiles [11].

The safety profile was one of several reasons that MVA-BN was nearly exclusively used during the mpox global outbreak. In humans, MVA-BN results in fewer adverse events compared to other smallpox vaccines [12,13,14]. In the US, 1.2 million doses of MVA-BN were administered between May 2022 and January 2023, but only 23% of the at-risk population was fully vaccinated [15]. MVA-BN is also being used for ongoing outbreaks in Africa [16]. It has been shown that MVA-BN induces low-level mpox-neutralizing antibody responses that wane within a year [17,18,19]. This is concerning, but anamnestic vaccinia-specific responses have been seen with low antibody levels or more than 2 years after vaccination, indicating that the type and levels of protective immunity are not fully known. Individuals who have a previous history of smallpox vaccination as part of the global eradication campaign are more protected against mpox compared to unvaccinated people, suggesting that replicating vaccines may have an mpox-protective capacity decades post-vaccination [20]. To our knowledge, there have been no studies on mpox immunity induced by replication-competent vaccines and it is not known if peak mpox-specific immune responses or the durability of mpox immunity of these vaccines is different from MVA-BN. This study was conducted with two objectives: (i) evaluate and compare the mpox cross-reactive neutralizing antibody responses induced by the MVA vaccine with the responses induced by a replication-competent vaccine, and (ii) evaluate and compare the mpox cross-reactive T cell responses induced by the MVA-BN vaccine with the responses induced by a replication-competent vaccine.

## 2. Materials and Methods

### 2.1. Study Design and Participants

Stored samples from three previously completed clinical trials were used [12,14,21]. All the trials were conducted on vaccinia-naïve participants. The age and sex of the participants in these studies were similar (Supplementary Table S1). One of the trials (NCT00914732) studied the effects of routes of administration on the immunity induced by two doses of MVA-BN injected 28 days apart [14]. Two doses of MVA were administered at 1 × 10^8^ TCID_50_ vaccine virus in 0.5 mL subcutaneously (SC) into the deltoid area, or 2 × 10^7^ tissue culture infectious dose (TCID)_50_ in 0.1 mL intradermally (ID) in the volar area of the forearm, 28 days apart. In this study, the study visits included pre-vaccination, days 14 and 28 post-first vaccination, and days 14, 28, and 180 post-second vaccination. The other trials used a replication-competent smallpox vaccine, Dryvax (NCT00082446 and NCT00437021) [12,21]. Dryvax was administered percutaneously (scarification) using 15 punctures with a sterile bifurcated needle containing a drop (approximately 0.0025 mL) of a vaccine reconstituted at a concentration of 1 × 10^8^ plaque-forming units (PFUs)/mL over the deltoid area. For NCT00082446, the study visits included pre-vaccination and days 4, 8, 14, 28, 180, and 365 post-vaccination. For NCT00437021, the study visits included pre-vaccination and day 70 post-vaccination. A total of 207 sera from the MVA-BN study and 95 sera from the Dryvax studies, which were collected before and at different time points after vaccination, were used for neutralization assays. A total of 192 PBMCs from the MVA-BN study and 52 PBMCs from the Dryvax studies, collected before and at different time points after vaccination, were used for T cell assays. Only samples from volunteers who consented to the future use of specimens were used after obtaining approval from the institutional review board. This study was conducted in accordance with the Declaration of Helsinki and approved by the Institutional Review Board (or Ethics Committee) of Saint Louis University.

### 2.2. Viruses

Clade II mpox (NR-2500, BEI Resources, MD, USA) and vaccinia (Dryvax, Wyeth Laboratories, Inc., PA, USA) were grown in BSC40 cells. The viral titers were quantified using a plaque assay and aliquots were stored at −80 °C. On the day of the experiments, the viral aliquots were thawed at room temperature, vortexed, and sonicated in a water bath sonicator for 15 s before they were used to prepare appropriate dilutions [22]. For some experiments, the mpox virus and vaccinia were UV-inactivated as described previously [22]. Inactivation was confirmed by a plaque assay of undiluted virus aliquots. Inactivated virus aliquots did not cause plaque formation.

### 2.3. Mpox Focus Reduction Neutralization Test (FRNT)

The FRNT was performed as described previously [22]. Briefly, different dilutions of heat-inactivated sera were mixed with an equal volume of a clade II mpox virus suspension in 96-well plates with a viral concentration of 150 PFUs per well and incubated at 37 °C overnight (8–10 h). Then, the serum–virus mixtures were transferred to new correspondingly labelled 96-well plates containing near-confluence BSC40 cells. After 1 h of incubation at 37 °C, a methylcellulose solution was added into each well and the plates were incubated for 24 h. Then, the cells were fixed with 5% paraformaldehyde and stained with A29 primary monoclonal antibodies (Sino Biological), followed by staining with horseradish peroxidase-labelled secondary antibodies (Invitrogen) and the addition of peroxidase substrate (Trublue, Sigma, Saint Louis, MO, USA). The number of spots were counted using a CTL ImmunoSpot analyzer. The FRNT50 was defined as the highest serum dilution that decreased the number of focus-forming units by at least 50% compared to serum-free controls.

### 2.4. Vaccinia Plaque Reduction Neutralization Test (PRNT)

The plaque reduction neutralization test (PRNT) was performed as described previously [23,24]. Briefly, serial two-fold dilutions of heat-inactivated sera were prepared. Each sera dilution was mixed with an equal volume of vaccinia virus (Dryvax) that had been freshly sonicated and approximately contained 40 to 60 PFUs of virus. The serum–virus mixtures were incubated overnight at 37 °C with 5% CO_2_. The mixtures were transferred to duplicate wells of a 24-well tissue culture plate containing a confluent monolayer of BSC40 cells and incubated for about 30 h at 37 °C with 5% CO_2_. Plaques were visualized and counted by staining monolayers with 0.1% crystal violet for five minutes. The PRNT50 was determined as the dilution resulting in at least a 50% reduction in the total plaques.

### 2.5. IFN-γ ELISPOT

The IFN-γ ELISPOT assay was performed according to the manufacturer’s instructions, using a kit from BD Bioscience as described previously [22]. Briefly, an anti-IFN-γ capture antibody in coating buffer was added to each well of a 96-well plate and incubated at 4 °C overnight. The plates were washed and PBMCs (3 × 10^5^ per well) were added into each well, followed by the addition of antigens, such as UV-inactivated clade II mpox, UV-inactivated vaccinia, or phorbol 12-myristate 13-acetate (PMA, Sigma, Saint Louis, MO, USA). PMA at 10 µg/mL was used as a positive control, and Dulbecco’s modified Eagle medium (Sigma, MO, USA) alone was used as a negative control. The tests were performed in triplicate. After 24 h of incubation at 37 °C, the plates were washed, biotin-conjugated anti-IFN-γ was added, and the plates were incubated for 2 h at room temperature. Then, the plates were washed, streptavidin–horseradish peroxidase solution was added, and the spots were counted using the Immunospot Micro Analyzer.

### 2.6. Statistical Analysis

The geometric mean titer (GMT), geometric mean fold rise (GMFR), and geometric mean titer ratio (GMTR) with a 95% confidence interval (CI) were used to analyze the FRNT50 and PRNT50 data. The GMFR represents the geometric mean fold rise in the antibody titer compared to the pre-vaccination titer. The GMTR is the ratio of GMT in MVA-BN recipients to Dryvax recipients. The GMFR was calculated for all post-vaccination time points. The GMTR was calculated for all time points following the second dose of MVA-BN. The CI was calculated using the Welch–Satterthwaite *t*-test. The peak response, the maximum response for each participant across all study visits, was obtained. Spearman’s correlation coefficient was used to analyze differences in the levels of mpox-specific and vaccinia-specific neutralizing antibody responses induced by MVA-BN and Dryvax. The medians with the minimum and maximum numbers of spot-forming cells (SFCs), the means with 95% CIs, the mean differences from baseline, and the difference in means with 95% CIs were used to compare the IFN-γ ELISPOT results. A 95% CI for the number of SFCs was calculated via percentile bootstrapping using 5000 samples. The difference in means for SFCs in the samples from MVA-BN recipients was calculated using the mean of the corresponding post-Dryvax vaccination time point.

The T cell response half-life was calculated as the time in days from the visit day with a peak response post-full dose to the first visit day with 50% of the peak response or less. Peak responses that occurred prior to the final dose for MVA were imputed as the visit day of the final dose. If the first day at or below 50% of the peak response occurred prior to the final dose of MVA, it was imputed as the visit day of the final dose.

## 3. Results

**Figure 1:**
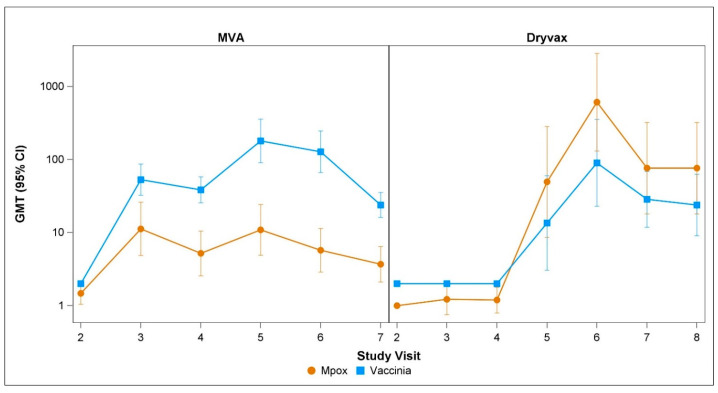
Changes in mpox- and vaccinia-specific neutralizing antibody titers following MVA and Dryvax vaccinations. The panel shows the GMT (95% CI) of the mpox-specific FRNT50 and vaccinia-specific PRNT50 for all MVA (i.e., subcutaneous and intradermal groups combined) and Dryvax vaccinees. The brown lines are for mpox-specific titers and the blue lines are for vaccinia-specific titers. Study visits 2, 3, 4, 5, 6, and 7 for MVA correspond to the pre-vaccination, day 14 post-first vaccination, day 28 post-first vaccination, day 14 post-second vaccination, day 28 post-second vaccination, and day 180 post-second vaccination time points, respectively. Study visits 2, 3, 4, 5, 6, 7, and 8 for Dryvax correspond to pre-vaccination and post-vaccination days 4, 8, 14, 28, 180, and 365, respectively.

**Figure 2:**
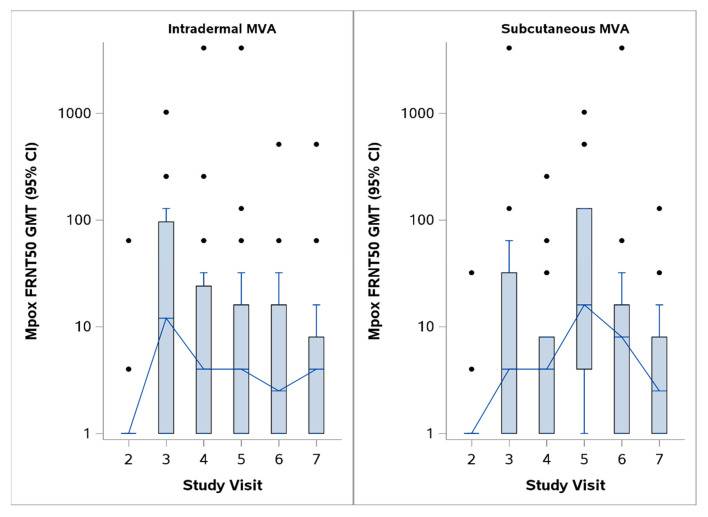
Mpox-specific FRNT50 for intradermal and subcutaneous MVA-BN. Intradermal and subcutaneous MVA induced mpox-specific neutralizing antibody responses, but the levels were lower than the peak mpox-specific neutralizing antibody responses induced by Dryvax and the peak vaccinia-specific neutralizing antibody responses induced by MVA-BN. The study visits 2, 3, 4, 5, 6, and 7 correspond to pre-vaccination, day 14 post-first vaccination, day 28 post-first vaccination, and days 14, 28, and 180 post-second vaccination.

Changes in the mpox-and vaccinia-specific neutralizing antibody responses following the first and second doses of MVA-BN are shown in Figure 1 and Figure 2. Figure 1 compares the data from all the MVA-BN participants (i.e., subcutaneous and intradermal MVA-BN groups combined) with the Dryvax group. In the MVA-BN recipients, the vaccinia-specific neutralizing antibody responses were significantly higher than the mpox-specific neutralizing antibody responses at all post-vaccination time points, including day 180 post-vaccination. There were fewer Dryvax recipients, but in this group, the vaccinia-specific and mpox-neutralizing responses overlapped except on day 28 post-vaccination, when significantly better mpox responses were seen. Figure 2 shows the mpox-specific neutralizing antibody responses to MVA-BN with SC administration and MVA-BN with ID administration separately. The mpox-specific neutralizing titers peaked on day 14 after the first dose of ID MVA-BN and 14 days after the second dose of SC MVA-BN. The vaccinia-specific responses peaked on day 14 after two doses of MVA-BN and on day 28 after the Dryvax vaccination. Spearman’s correlation for the mpox- and vaccinia-specific neutralizing antibody responses induced by MVA-BN (i.e., values for subcutaneous and intradermal groups combined) was 0.54, 0.44, and 0.32 on days 14, 28, and 180 post-vaccination, respectively. Spearman’s correlation for the mpox-and vaccinia-specific neutralizing antibody responses induced by Dryvax was 0.78, 0.17, and 0.44 on days 14, 28, and 180 post-vaccination, respectively (Table 1).

MVA-BN induced significantly lower levels of mpox-specific neutralizing antibodies compared to the replication-competent smallpox vaccine. Table 2 shows the GMT, GMTR, GMFR, and peak responses post-vaccination with MVA-BN and Dryvax. The peak GMFR (95% CI), which was the maximum response across all study visits for MVA-BN SC, MVA-BN ID, and Dryvax, was 22.1 (8.3, 59.1), 18.5 (8.0, 43.1), and 245.8 (100.4, 601.6), respectively. The GMFR (95% CI) of MVA-BN-induced mpox-specific FRNT50 titers was 15.2 (4.7, 49.0) and 4.5 (2.1, 9.5) on day 14 following subcutaneous and intradermal administration, respectively. The GMFR (95% CI) of the mpox-specific FRNT50 decreased to 2.4 (1.1, 5.4) and 2.7 (1.5, 4.9) on day 180 after two doses of SC MVA-BN and ID MVA-BN, respectively. The GMTR (i.e., the ratio of the GMT for MVA-BN recipients to that for Dryvax recipients) after two MVA-BN doses was 0.2, 0, and 0 on days 14, 18, and 180 post-full vaccination, respectively. These indicate that mpox-FRNT50 titers were induced by MVA-BN at much lower levels than those induced by the replication-competent smallpox vaccine (Dryvax).

The medians, means (95% CI), and mean differences (95% CI) of the mpox-specific T cell responses for pre- and post-vaccination samples are shown in Table 3. The mean (95% CI) peak number of mpox-specific IFN-γ SFCs was 127 (43.1, 238.3), 87.3 (46, 137), and 61.2 (44.3, 77.7) following two doses of subcutaneous MVA-BN, intradermal MVA-BN, or Dryvax, respectively. On day 14 post-full dose, the mean differences from baseline (95% CI) were 50 (22.5, 83.6) and 38.5 (12.5, 65.7) for all MVA-BN and Dryvax samples, respectively. On day 180 following full doses of vaccination, the values decreased to 22.5 (−15.2, 8.7) and 2.2 (−9, 12.5), respectively. On day 365, the mean difference from baseline (95% CI) for Dryvax recipients was 19.1 (−3.7, 44.5). The levels for the vaccinia-specific T cell responses were similar to the mpox-specific T cell responses (Supplementary Table S2).

We also analyzed the decline rates in T cells between MVA and Dryvax vaccinees by calculating half-lives. Table 4 shows that the half-life of the mpox T cell responses was longer for Dryvax vaccinees, with a mean (95% CI) half-life of 88.3 days (33, 143.5) and 20 days (7, 37.1) for Dryvax and MVA, respectively.

**Figure 3:**
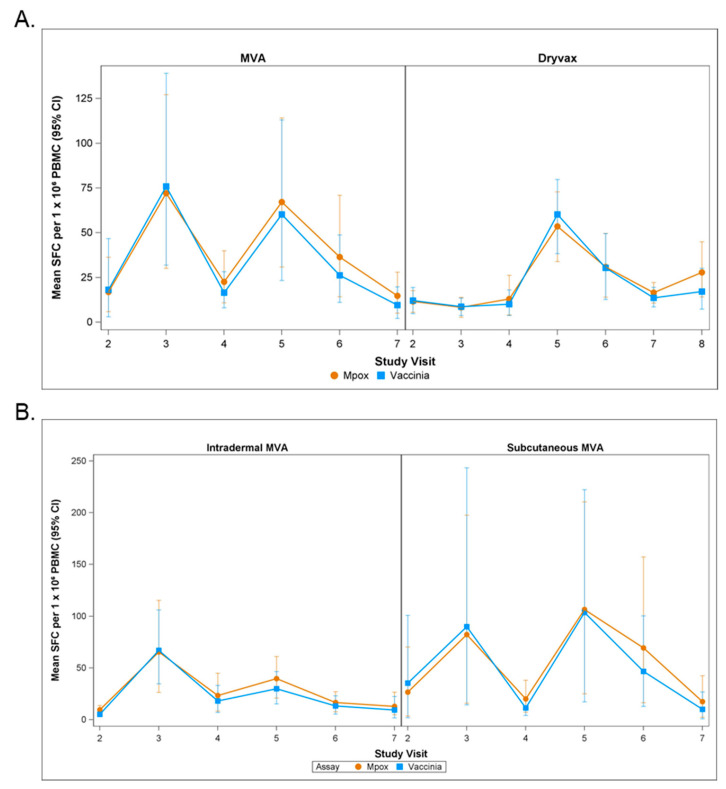
Changes in mpox- and vaccinia-specific T cell responses following MVA and Dryvax vaccinations. Panel (**A**) shows mpox (brown line)- and vaccinia (blue line)-specific T cell responses following MVA and Dryvax vaccinations. Study visits 2, 3, 4, 5, 6, and 7 for MVA correspond to pre-vaccination and the day 14 post-first vaccination, day 28 post-first vaccination, day 14 post-second vaccination, day 28 post-second vaccination, and day 180 post-second vaccination time points, respectively. Study visits 2, 3, 4, 5, 6, 7, and 8 for Dryvax correspond to pre-vaccination and days 4, 8, 14, 28, 180, and 365 post-vaccination, respectively. Following MVA vaccination, the mean numbers of mpox-specific and vaccinia-specific IFN-γ SFCs were highest on day 14 post-first and post-second vaccination, but rapidly decreased on day 28 following the first vaccination or day 180 following the second vaccination. Following Dryvax vaccination, the mean numbers of mpox-specific and vaccinia-specific IFN-γ SFCs were highest on day 14 post-vaccination and gradually declined to lower levels on days 180–365. Panel (**B**) shows the changes in the mpox- and vaccinia-specific T cell responses for intradermal MVA and subcutaneous MVA separately.

Changes in mpox- and vaccinia-specific T cell responses following the first and second doses of MVA-BN are shown in Figure 3. There were wide variations in the responses. On day 14 post-first dose, the mean (95% CI) for subcutaneous MV-BN and intradermal MVA-BN was 89.9 (14.4, 233.7) and 67 (34.5, 106.5), respectively. On day 14, the mean (95% CI) for the second dose of subcutaneous MV-BN, second dose of intradermal MVA-BN, and Dryvax was 103.6 (17.1, 219.3), 29.9 (15.1, 46.2), and 60.2 (38.4, 79.8), respectively.

## 4. Discussion

This study showed that a replication-competent smallpox vaccine induced higher levels of mpox-specific neutralizing antibody titers (i.e., 10 times higher peak responses) compared to MVA-BN, a non-replicating smallpox vaccine. It was recently shown that mpox immunity correlates with antigenic distance [25]. Other studies have shown that MVA-BN induces low levels of mpox-specific neutralizing antibody titers, but comparative studies are lacking [17,26]. The reasons why MVA-BN induces lower mpox-neutralizing antibody responses while inducing similar levels of vaccinia-neutralizing antibody responses to Dryvax are not known. MVA-BN has lost some genes during hundreds of passages, which has made it lose its ability to replicate in mammalian cells [27,28]. The importance of genes deleted from MVA on the overall mpox immunity remains to be studied.

The mpox-specific neutralizing antibody responses induced by MVA-BN decreased markedly at day 180 after the second dose. This is similar to findings obtained by others [29]. In our study, the kinetics of mpox-specific neutralizing antibody responses were similar between ID and SC MVA-BN. Interestingly, the mpox-specific neutralizing antibody responses induced by a replication-competent smallpox vaccine also declined markedly at day 180 post-vaccination. However, the level of mpox-neutralizing antibody responses at day 180 was higher with a replication-competent vaccine compared to MVA-BN (GMFR 2.6 vs. 64). Some assume that levels below 1 in 10 or 1 in 20 may indicate seronegative results [30,31,32], but findings prior to the eradication of smallpox indicate that vaccinations protected against smallpox, despite them resulting in neutralizing antibody titers below 1 in 10 [33]. In addition, recent findings suggest that a low level of MVA-BN-induced vaccinia-specific immunity two years post-vaccination could be boosted to a level seen in individuals with a history of vaccination with a replication-competent vaccine [34]. Therefore, further studies are needed to determine if the low-level residual mpox immunity several months or years after MVA-BN vaccination is protective.

Studies in nonhuman primates have shown that T cells are important for mpox protection [35]. CD4 T cells are needed for immunoglobulin switching, and therefore, they are key for optimal functional neutralizing antibody responses [35]. These results were confirmed by findings from the recent global mpox outbreak, where it was shown that the number of CD4 T cells predicted the risk of hospitalization and death from mpox [36]. Our results, as shown by our IFN-γ ELISPOT assays, indicate that MVA-BN induces T cell responses at a level similar to the responses induced by a replication-competent vaccine. The levels of mpox-specific T cell responses were similar to the levels of vaccinia-specific T cell responses induced by MVA-BN. In addition, the maximum numbers of SFC/10^6^ PBMCs post-vaccination were similar for MVA-BN and the replication-competent vaccinia vaccine. However, the mpox T cell responses induced by the replication-competent vaccine were more sustained compared to the T cell responses induced by MVA-BN, with more than 4-fold differences in the half-life. In MVA-BN vaccinees, the T cell responses decreased markedly close to the pre-vaccination level at day 180 post-vaccination.

Contrary to our findings, one study reported that MVA-BN-induced T cell responses remained high at 12 months post-vaccination [29]. A volunteer in the subcutaneous MVA-BN study group had a high pre-vaccination T cell response, but the reason for this was not clear, although subclinical exposure to pox viruses is a possibility.

The limitation of this study is that it was performed on stored samples from previous clinical trials. The samples were stored under optimal conditions. The participants in the MVA-BN and Dryvax studies had similar age and sex distributions. The number of participants in the different groups was different because different numbers of participants were included in these studies. The small sample size of Dryvax recipients limited the statistical power and generalizability. The number of participants who provided pre-Dryvax vaccination samples was higher than the number who provided post-vaccination samples. This was because 11 samples from a second Dryvax study, which had only two time points (i.e., pre-vaccination and day 70 post-vaccination), were used. In addition, the figures and tables show wide 95% CIs, and this could be due to the small sample size of the vaccination groups and a few outliers with a markedly high response at different time points, including a pre-vaccination time point. Despite the limitation, this study was able to compare mpox-specific and vaccinia-specific neutralizing antibody and T cell responses between MVA-BN recipients and recipients of a replication-competent vaccine.

## 5. Conclusions

Our study shows that MVA-BN induced mpox-specific neutralizing antibody responses, but the levels were much lower compared to the levels induced by a replication-competent smallpox vaccine. This was unlikely to be due to the antigen amount, because MVA-BN induced vaccinia-specific neutralizing antibody responses with levels much higher than the levels obtained following vaccination with the replication-competent smallpox vaccine. The MVA-BN-induced mpox-neutralizing antibody titers waned rapidly, with levels close to baseline at day 180 post-vaccination. The effects of low-level mpox-neutralizing antibody responses and their rapid decline on mpox protection are not known. It is important to determine if residual mpox immune responses at 6 months or longer after MVA-BN vaccination are protective. MVA-BN induces similar levels of T cell responses as a replication-competent smallpox vaccine, but these responses wane markedly at day 180 post-vaccination. These results will help policy makers make an informed decision on the potential benefits relative to the potential risks of using these vaccines during mpox outbreaks.

## Figures and Tables

**Table 1:** Comparison of mpox- and vaccinia-neutralizing antibody responses.

		MVA		Dryvax	
Time Point	Statistic	Mpox	Vaccinia	Mpox	Vaccinia
Visit 2 (pre-dose 1)	N	34	34	6	6
	GMT (95% CI) ^a^	1.5 (1.0, 2.1)	2.0 (NE)	1.0 (1.0, 1.0)	2.0 (2.0, 2.0)
	Spearman’s correlationcoefficient	--	NE	--	NE
Day 14 (13–15) MVA post-dose 2vs. day 14 (13–15) post-Dryvax	N	34	34	8	8
	GMT (95% CI) ^a^	10.9 (4.9, 24.2)	179.3 (90.3, 355.9)	49.4 (8.6, 284.1)	13.5 (3.1, 59.9)
	Spearman’s correlationcoefficient	--	0.54	--	0.78
Day 28 (26–30) MVA post-dose 2vs. day 28 (26–30) post-Dryvax	N	35	35	8	8
	GMT (95% CI) ^a^	5.7 (2.9, 11.3)	127.3 (66.0, 245.5)	608.9 (130.4, 2842.9)	90.0 (22.9, 353.4)
	Spearman’s correlationcoefficient	--	0.44	--	0.17
Day 180 (177–183) MVA post-dose 2vs. day 180 (177–183) post-Dryvax	N	34	34	8	8
	GMT (95% CI) ^a^	3.7 (2.1, 6.5)	23.8 (16.1, 35.3)	76.1 (18.0, 322.7)	28.4 (11.8, 68.7)
	Spearman’s correlationcoefficient	--	0.32	--	0.44
Day 365 (351–379) post-Dryvax	N	--	--	8	8
	GMT (95% CI) ^a^	--	--	76.1 (18.0, 322.7)	23.8 (9.0, 62.8)
	Spearman’s correlationcoefficient	--	--	--	0.24
Peak response ^b^	N	35	35	8	8
	GMT (95% CI) ^a^	30.8 (14.6, 65.0)	266.1 (156.0, 453.9)	789.6 (238.5, 2614.1)	123.7 (37.6, 406.7)
	Spearman’s correlationcoefficient	--	0.26	--	0.11

Only participants with both FRNT50 and PRNT data at each time point were included. N, number of participants with data at the given time point. CI, confidence interval. NE, not estimable. ^a^ CIs were calculated based on Student’s t distribution. ^b^ The peak response is the maximum response for each participant across all study visits.

**Table 2:** Mpox-neutralizing titers induced by MVA-BN or Dryvax.

Time Point	Statistic	All MVA	MVA Subcutaneous	MVA Intradermal	Dryvax
Visit 2 (pre-dose 1)	N	34	15	19	17
	GMT (95% CI) ^a^	1.5 (1.0, 2.1)	1.4 (0.8, 2.3)	1.5 (0.9, 2.6)	1.4 (0.9, 2.1)
	GMTR ^c^ (95% CI) ^a^	1.5 (1.0, 2.1)	1.4 (0.8, 2.3)	1.5 (0.9, 2.6)	--
Day 14 (13–15) MVA post-dose 2vs. day 14 (13–15) post-Dryvax	N	34	14	20	8
	GMT (95% CI) ^a^	10.9 (4.9, 24.2)	21.5 (5.3, 87.5)	6.7 (2.5, 18.1)	49.4 (8.6, 284.1)
	GMTR ^c^ (95% CI) ^a^	0.2 (0.0, 1.4)	0.4 (0.1, 3.5)	0.1 (0.0, 0.9)	--
	GMFR ^b^ (95% CI) ^a^	7.5 (3.9, 14.5)	15.2 (4.7, 49.0)	4.5 (2.1, 9.5)	22.6 (3.6, 140.5)
Day 28 (26–30) MVA post-dose 2vs. day 28 (26–30) post-Dryvax	N	35	15	20	8
	GMT (95% CI) ^a^	5.7 (2.9, 11.3)	8.8 (2.6, 30.0)	4.1 (1.8, 9.6)	608.9 (130.4, 2842.9)
	GMTR ^c^ (95% CI) ^a^	0.0 (0.0, 0.0)	0.0 (0.0, 0.1)	0.0 (0.0, 0.0)	--
	GMFR ^b^ (95% CI) ^a^	4.1 (2.4, 7.0)	6.3 (2.6, 15.7)	2.9 (1.4, 5.8)	645.1 (68.8, 6048.0)
Day 180 (177–183) MVA post-dose 2vs. day 180 (177–183) post-Dryvax	N	34	14	20	8
	GMT (95% CI) ^a^	3.7 (2.1, 6.5)	3.4 (1.4, 8.5)	3.9 (1.8, 8.5)	76.1 (18.0, 322.7)
	GMTR ^c^ (95% CI) ^a^	0.0 (0.0, 0.2)	0.0 (0.0, 0.2)	0.1 (0.0, 0.2)	--
	GMFR ^b^ (95% CI) ^a^	2.6 (1.6, 4.1)	2.4 (1.1, 5.4)	2.7 (1.5, 4.9)	64.0 (7.8, 527.0)
Day 365 (351–379) post-Dryvax	N	--	--	--	8
	GMT (95% CI) ^a^	--	--	--	76.1 (18.0, 322.7)
	GMFR ^b^ (95% CI) ^a^	--	--	--	57.0 (8.4, 388.9)
Peak response ^d^	N	35	15	20	19
	GMT (95% CI) ^a^	30.8 (14.6, 65.0)	30.6 (8.6, 108.7)	30.9 (11.3, 84.6)	368.7 (188.4, 721.7)
	GMTR ^c^ (95% CI) ^a^	0.0 (0.0, 0.1)	0.0 (0.0, 0.2)	0.0 (0.0, 0.2)	--
	GMFR ^b^ (95% CI) ^a^	20.0 (10.9, 36.7)	22.1 (8.3, 59.1)	18.5 (8.0, 43.1)	245.8 (100.4, 601.6)

N, number of participants with data at the given time point. CI, confidence interval. ^a^ CIs were calculated based on Student’s t distribution. ^b^ GMFR represents the geometric mean fold rise in the antibody titer compared to pre-dose 1. ^c^ GMTR is the ratio of the geometric mean titer in MVA-BN recipients to Dryvax recipients. ^d^ The peak response is the maximum response for each participant across all study visits.

**Table 3:** Comparisons of numbers of mpox-specific IFN-γ spot-forming T cells induced by MVA-BN or Dryvax.

Time Point	Statistic	All MVA	MVA Subcutaneous	MVA Intradermal	Dryvax
Visit 2 (pre-dose 1)	N	35	15	20	6
	Median (min, max)	4.4 (0, 325.6)	4.4 (0, 325.6)	7.2 (0, 34.1)	9.9 (0, 23.1)
	Mean (95% CI) ^a^	16.8 (5.8, 36.3)	26.5 (3.5, 70.5)	9.6 (5.8, 13.8)	11.6 (5.5, 17.6)
	Difference in means (95% CI) ^a^	5.3 (−8.9, 27.1)	15 (−11.1, 61.1)	−2 (−9.1, 5.3)	--
Day 14 (13–15) MVA post-dose 2vs. day 14 (13–15) post-Dryvax	N	34	14	20	8
	Median (min, max)	13.8 (0, 613.8)	13.8 (1.1, 613.8)	14.9 (0, 158.4)	51.2 (14.3, 90.2)
	Mean (95% CI) ^a^	67.2 (31.1, 115.4)	106.5 (25.7, 208.4)	39.8 (20.6, 61.1)	53.6 (33.9, 72.9)
	Mean difference from baseline (95% CI) ^a^	50 (22.5, 83.6)	78.2 (19.9, 147.5)	30.2 (11.2, 50.9)	38.5 (12.5, 65.7)
	Difference in means (95% CI) ^a^	13.6 (−27.7, 63.4)	52.8 (−31.9, 158.1)	−13.9 (−41, 15.1)	--
Day 28 (26–30) MVA post-dose 2vs. day 28 (26–30) post-Dryvax	N	32	12	20	8
	Median (min, max)	8.3 (0, 511.5)	23.7 (0, 511.5)	5 (0, 77)	17.6 (0, 73.7)
	Mean (95% CI) ^a^	36.4 (14.4, 71.6)	69.4 (15.8, 155.7)	16.6 (7.8, 26.9)	30.9 (14, 49.6)
	Mean difference from baseline (95% CI) ^a^	18.1 (5.7, 33.6)	36.7 (9.5, 71)	7 (−2.5, 17.3)	26 (3.7, 49.9)
	Difference in means (95% CI) ^b^	5.4 (−25.3, 44)	38.5 (−20, 127.7)	−14.4 (−35.6, 5.6)	--
Day 180 (177–183) MVA post-dose 2vs. day 180 (177–183) post-Dryvax	N	34	14	20	8
	Median (min, max)	3.9 (0, 172.7)	2.2 (0, 172.7)	4.7 (0, 129.8)	19.3 (5.5, 25.3)
	Mean (95% CI) ^a^	14.7 (5.1, 28.6)	17.4 (2.6, 42.5)	12.9 (4.8, 26.8)	16.5 (10.7, 22.1)
	Mean difference from baseline (95% CI) ^a^	−2.5 (−15.2, 8.7)	−10.8 (−34.4, 3.8)	3.3 (−6.2, 18.6)	2.2 (−9, 12.5)
	Difference in means (95% CI) ^b^	−1.8 (−13.1, 12.6)	0.9 (−16.2, 27.7)	−3.6 (−14.8, 11.5)	--
Day 365 (351–379) post-Dryvax	N	--	--	--	8
	Median (min, max)	--	--	--	23.7 (0, 79.2)
	Mean (95% CI) ^a^	--	--	--	27.9 (14.1, 45)
	Mean difference from baseline (95% CI) ^a^	--	--	--	19.1 (−3.7, 44.5)
Peak response ^c^	N	35	15	20	8
	Median (min, max)	38.5 (0, 727.1)	25.3 (4.4, 727.1)	39.1 (0, 424.6)	68.8 (23.1, 90.2)
	Mean (95% CI) ^a^	104.3 (61, 158.7)	127 (43.1, 238.3)	87.3 (46, 137)	61.2 (44.3, 77.7)
	Mean difference from baseline (95% CI) ^a^	87.5 (51.8, 129.1)	100.5 (38.7, 172.5)	77.7 (35.9, 127.9)	48.6 (22.7, 70.8)
	Difference in means (95% CI) ^b^	43.1 (−4.8, 98.6)	65.8 (−16.8, 178.8)	26.1 (−18.6, 78.8)	--

N, number of participants with data at the given time point. SFCs, spot-forming cells. CI, confidence interval. The difference in means was calculated as the mean MVA-BN response—mean Dryvax response. ^a^ CIs were calculated via percentile bootstrapping using 5000 samples. ^b^ The difference in means was calculated using the mean of the corresponding day post-dose 1 for 06-0012. ^c^ The peak response is the maximum response for each participant across all study visits.

**Table 4:** Half-life of immune response in days, as measured by IFN-γ spot-forming T cells induced by MVA-BN or Dryvax.

Statistic	All MVA	MVA Subcutaneous	MVA Intradermal	Dryvax
N	34	14	20	8
Median (min, max)	0 (0, 166)	14 (0, 166)	0 (0, 166)	83 (14, 166)
Mean (95% CI) ^a^	20 (7, 37.1)	29.7 (6, 61.3)	13.2 (2.8, 30.5)	88.3 (33, 143.5)
Difference in means (95% CI) ^a^	−68.3 (−121.5, −15.4)	−58.5 (−117.3, 0.4)	−75.1 (−126.9, −21.5)	--

N, number of participants with data at the given time point. CI = confidence interval. The difference in means was calculated as the mean MVA response–mean Dryvax response. ^a^ CIs were calculated via percentile bootstrapping using 5000 samples.

## Data Availability

The research data are available from the corresponding author upon request.

## Supplementary Materials

The following supporting information can be downloaded at: https://www.mdpi.com/article/10.3390/vaccines13050520/s1, Table S1: Age and sex of participants in the MVA-BN and Dryvax studies; Table S2: Comparisons of numbers of vaccinia-specific IFN-γ spot forming T cells induced by MVA-BN and Dryvax.
