# Supplementary material for: Differences in Mpox and Vaccinia Immunity Induced by Non-Replicating and Replicating Vaccinia-Based Vaccines
Source: Vaccines (Basel). 2025 May 14;13(5):520. doi: 10.3390/vaccines13050520 (PMC12115885; doi:10.3390/vaccines13050520)
Supplement: Supplementary file 1 [file vaccines-13-00520-s001.zip › vaccines-3566792-supplementary.pdf]

**Supplementary Table S1.** Age and sex of participants in the MVA-BN and Dryvax studies.

| Characteristic (unit), Statistic | MVA Subcutaneous (N=15) | MVA Intradermal (N=20) | Dryvax (N=8) |
|---|---|---|---|
| **Sex, n (%)** | | | |
| Male | 6 (40) | 11 (55) | 7 (88) |
| Female | 9 (60) | 9 (45) | 1 (13) |
| **Age (years)** | | | |
| Mean (SD) | 27.3 (3.0) | 26.2 (4.8) | 26.6 (4.2) |
| Median | 28.0 | 26.0 | 26.0 |
| Min, Max | 23, 32 | 20, 37 | 20, 32 |

Note: N=Number of participants with demographic data available.

**Supplementary Table S2. Comparisons of numbers of vaccinia-specific IFN-γ spot forming T cells induced by MVA-BN and Dryvax.**

| Time Point | Statistic | All MVA | MVA Subcutaneous | MVA Intradermal | Dryvax |
|---|---|---|---|---|---|
| **Visit 2 (Pre-Dose 1)** | N | 35 | 15 | 20 | 6 |
| | Median (min, max) | 3.3 (0, 489.5) | 3.3 (0, 489.5) | 2.8 (0, 23.1) | 12.1 (0, 26.4) |
| | Mean (95% CI)[a] | 18.1 (3, 46.9) | 35.3 (2, 100.9) | 5.2 (2.8, 8.1) | 12.1 (4.8, 19.4) |
| | Difference in Means (95% CI)[a] | 6 (-13.8, 38.1) | 23.2 (-15.2, 92.3) | -6.9 (-14.9, 1) | -- |
| **Day 14 (13-15) MVA post-dose 2 vs Day 14 (13-15) post-Dryvax** | N | 34 | 14 | 20 | 8 |
| | Median (min, max) | 8.3 (0, 750.2) | 5 (0, 750.2) | 15.7 (0, 124.3) | 71.5 (7.7, 93.5) |
| | Mean (95% CI)[a] | 60.2 (23.5, 114.2) | 103.6 (17.1, 219.3) | 29.9 (15.1, 46.2) | 60.2 (38.4, 79.8) |
| | Mean difference from Baseline (95% CI)[a] | 41.6 (19.2, 68.6) | 65.8 (14.8, 123.1) | 24.6 (10.4, 40.6) | 40.5 (15.7, 66.3) |
| | Difference in Means (95% CI)[a] | 0 (-43, 56.5) | 43.3 (-44.5, 162.1) | -30.4 (-55.3, -3.5) | -- |
| **Day 28 (26-30) MVA post-dose 2 vs Day 28 (26-30) post-Dryvax** | N | 31 | 12 | 19 | 8 |
| | Median (min, max) | 8.3 (0, 312.4) | 14.3 (0, 312.4) | 3.3 (0, 75.9) | 22.6 (0, 71.5) |
| | Mean (95% CI)[a] | 26.3 (11.1, 48.7) | 46.7 (12.7, 99.8) | 13.3 (5.6, 23.1) | 30.3 (12.7, 49.5) |
| | Mean difference from Baseline (95% CI)[a] | 5.9 (-9.5, 18.3) | 2.5 (-34.8, 29.2) | 8.1 (-0.8, 18.3) | 26.8 (5, 50.6) |
| | Difference in Means (95% CI)[a] | -4.1 (-29.2, 24.4) | 16.4 (-24.5, 73) | -17 (-37.5, 3.4) | -- |
| **Day 180 (177-183) MVA post-dose 2 vs Day 180 (177-183) post-Dryvax** | N | 34 | 14 | 20 | 8 |
| | Median (min, max) | 1.4 (0, 116.6) | 1.1 (0, 116.6) | 1.9 (0, 116.6) | 12.7 (3.3, 29.7) |
| | Mean (95% CI)[a] | 9.6 (2.1, 20) | 10.1 (0.9, 26.9) | 9.4 (1.7, 22.6) | 13.6 (8.5, 19.5) |
| | Mean difference from Baseline (95% CI)[a] | -9 (-34.8, 7.7) | -27.8 (-81.5, -0.1) | 4.1 (-4.6, 18.1) | -0.9 (-11.6, 10) |
| | Difference in Means (95% CI)[a] | -4 (-13.9, 7.6) | -3.6 (-16.1, 15) | -4.3 (-15, 9.8) | -- |
| **Day 365 (351-379) post-Dryvax** | N | -- | -- | -- | 8 |
| | Median (min, max) | -- | -- | -- | 9.9 (2.2, 56.1) |
| | Mean (95% CI)[a] | -- | -- | -- | 17.2 (7.3, 30.1) |
| | Mean difference from Baseline (95% CI)[a] | -- | -- | -- | 6.2 (-13.9, 29.3) |
| **Peak Response[c]** | N | 35 | 15 | 20 | 8 |
| | Median (min, max) | 38.5 (0, 846.5) | 26.4 (3.3, 846.5) | 47.9 (0, 303.6) | 71.5 (29.7, 93.5) |
| | Mean (95% CI)[a] | 93.2 (51.2, 150.4) | 116.1 (32.4, 243.7) | 76 (44, 112.9) | 65.2 (48.5, 81.1) |
| | Mean difference from Baseline (95% CI)[a] | 75.1 (45.7, 107.9) | 80.7 (30.3, 141.8) | 70.8 (38.9, 108) | 47.1 (27.3, 70) |
| | Difference in Means (95% CI)[a] | 28 (-18.5, 87.6) | 50.9 (-34.3, 177.1) | 10.9 (-25.7, 50.8) | -- |

N, number of participants with data at the given time point. SFC, spot-forming cells. CI, confidence interval.
Difference in means is calculated as mean MVA response – mean Dryvax response.

[a] CI calculated via percentile bootstrapping using 5,000 samples.

[b] Difference in means calculated using the mean of the corresponding day post dose 1 for 06-0012.

[c] Peak response is the maximum response for each participant across all study visits.
